# A new technique for nanoparticle transport and its application in a novel nano-sieve

**DOI:** 10.1038/s41598-018-28033-5

**Published:** 2018-06-26

**Authors:** Shuai Wang, Chao Wang, Zhilong Peng, Shaohua Chen

**Affiliations:** 1 LNM, Institute of Mechanics, Chinese Academy of Sciences, Beijing, 100190 China; 2 Institute of Advanced Structure Technology, Beijing Institute of Technology, Beijing, 100081 China; 3 Beijing Key Laboratory of Lightweight Multi-functional Composite Materials and Structures, Beijing Institute of Technology, Beijing, 100081 China; 4 School of Engineering Sciences, University of Chinese Academy of Sciences, Beijing, 100049 China

## Abstract

A new technique is proposed to transport and further classify nanoparticles of different sizes. A graphene sheet is used as the substrate; a nanoparticle is placed on the substrate and a sliding block is located below the substrate. As the sliding block moves under the graphene substrate, a driving force is yielded from the van der Waals interaction between the sliding block and the nanoparticle. The effects of the pre-tension of the graphene substrate, size and number of layers of the nanoparticle, slip velocity, the interface commensurability and temperature on nanoparticle transportation are systematically investigated. It is found that a pre-tensioned graphene substrate could provide easier nanoparticle transport. The initial movement of the nanoparticle depends on the competition between the in-plane force and the driving force, while the subsequent transport depends on the slip velocity of the sliding block and the viscous damping force. Based on such a new transport mechanism, a novel nano-sieve can be designed, with which nanoparticles of different sizes can be screened and classified spontaneously. Our findings may be useful for promising designs of transportation, manipulation and classification of nanoparticles.

## Introduction

Nanoscale manipulation refers to the controllable transportation, separation, and classification of nano-objects that can be further used to design more complex nano-devices and nano-instruments with nanoscale elements, and should be very useful for development in nano-science and nano-industries, for example, integrated DNA analysis devices^1^, lab-on-a-chip technologies^2^, nano-electromechanical devices^3,4^, and medical devices^5^.

Different interesting mechanisms or technologies have been proposed or used for nanoscale manipulation. Atomic force microscopy, scanning tunneling microscopy, frictional force microscopy, and micro-grippers^6^ have been generally adopted to manipulate objects as small as nanometers. To realize directional transport of micro- or nano-objects on a substrate, the substrate should have an inhomogeneous and gradient field, including the non-uniform properties of surface wetting^7–9^, surface curvature^10^, body temperature^11^, and material stiffness^12^. A non-uniformly tensile strain in the substrate induced by an external force can cause a small object to move or roll on its surface^13^. Sometimes, an external magnetic, electric, or even optical field can help to manipulate small objects. For example, an electric field can be used to trap or release charged gold nanoparticles onto or from the surface of gold electrodes^14^. Fennimore *et al*.^4^ constructed a fully synthetic electromechanical actuator by incorporating a rotatable metal plate with a multi-walled carbon nanotube serving as the key motion-enabling element. Alternating current dielectrophoresis could be used to control the deposition of single-walled carbon nanotubes^15^. A micro-electromagnet is an appropriate tool to precisely control and manipulate magnetic nanoparticles^16^. An optical tweezer is often adopted to trap dielectric nanoparticles, metal nanoparticles, or even DNA molecules^17,18^.

Some of these technologies are still at evolution and development phase and can only be performed at laboratory level, which have still a long way for industrial applications. Precisely manipulating small objects or particles remains a challenge, not to mention the classification problem of nanoparticles of different sizes.

Inspired by the free movement of small iron pieces on a flat piece of paper using a magnet lying under the paper, we propose a similar technique to manipulate nanoparticles. We adopt a similar system in which nanoparticles lie on a graphene sheet with a sliding block underneath. The movement of sliding block under the graphene sheet causes the movement of particles in the same direction. The difference is that the driving force in the first case is the well-known magnetic force, but the driving force in the second case is the familiar van der Waals interactions at a small scale. Based on this proposed manipulation technique, a novel nano-sieve is designed to screen and classify nanoparticles of different sizes.

Such a system could be set up experimentally considering the related skills and methods. The synthetic methods for producing a single-layer graphene sheet are relatively mature^19^. Techniques to manipulate a graphene sheet have also been studied, for example, by tailoring a graphene sheet to a certain shape and size^20^, stretching or bending a graphene sheet^21^, and connecting a graphene sheet to a carbon nanotube^22^ or a metal block^23^. Researchers have studied the motion of nanoflakes on graphene, including superlubric sliding^24^ and self-retracting motion^25^. All these skills enable the construction of a more complex system to realize various functions.

The outline of this paper is as follows. We first establish the manipulation system and find the transport phenomenon studied in the present paper. Then, the effects of the particle size, pre-tension strain in the graphene sheet, slip velocity of the sliding block, damping force, temperature and initial interface commensurability between the nanoparticle and substrate on the driving mechanism are investigated thoroughly. Based on such a driving mechanism, a novel sieve is then designed to classify nanoparticles of different sizes.

## Results

### Simulation model and methodology

**Figure 1:**
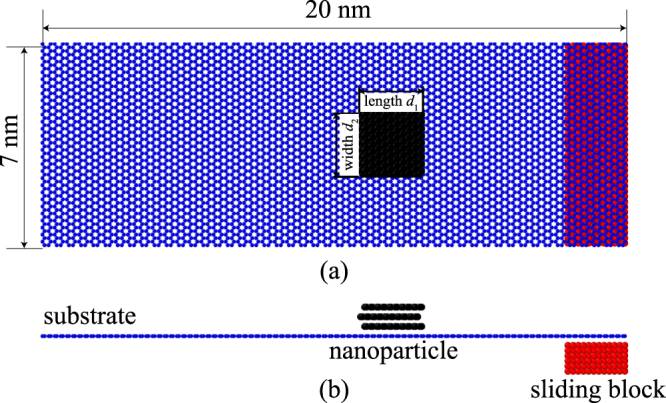
Schematic of the simulation system. (**a**) Top view and (**b**) front view.

A molecular dynamics simulation method is used in this paper to study the transport of nanoparticles driven by van der Waals forces. Figure 1 shows a representative numerical model. The nanoparticle is represented by a rectangular three-layered graphene sheet as an example, which is placed on a relatively large single-layer graphene sheet substrate (20 nm × 7 nm) with an *AB* staking structure. The length (*d*_1_) and width (*d*_2_) of the nanoparticle can be changed in detailed simulations, whose effects on the transportation mechanism are investigated. The side length (*d*) of a nanoparticle is further defined as the average value of its length and width for an approximately square nanoparticle. A sliding block is placed under the graphene sheet substrate. The sliding block is simulated using a copper material as an example. Considering the possible resistance exerted by the substrate in real applications (e.g., the resistance induced by the pre-existing wrinkles or roughness), we assume a viscous damping force ${F}_{{\rm{v}}{\rm{i}}{\rm{s}}}$ on each atom of the nanoparticle that is proportional to the nanoparticle’s velocity ${{v}}_{{\rm{P}}}$, i.e., ${{F}}_{{\rm{v}}{\rm{i}}{\rm{s}}}={\gamma }{{v}}_{{\rm{P}}}$, to dissipate the kinetic energy of a moving nanoparticle, where $\gamma$ denotes the viscous damping coefficient. Except for special instructions, all simulations take the value *γ* = 0.03 eVps/nm^2^.

### Transport mechanism and its influencing factors

**Figure 2:**
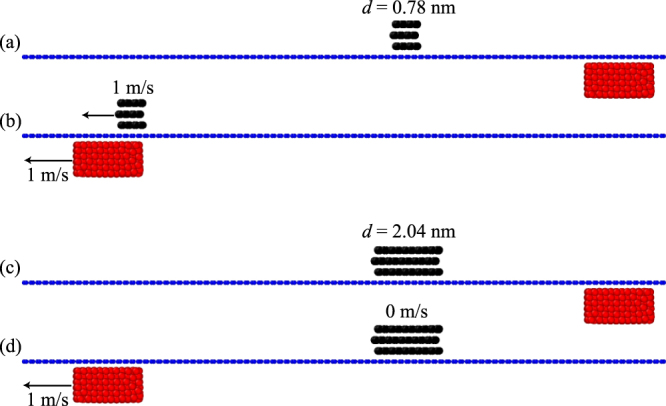
Typical snapshots of a nanoparticle on a substrate driven by the underneath sliding block at different sliding velocities.

In the present paper, the static friction between the nanoparticle and graphene substrate is called an in-plane force. The dynamic friction between the nanoparticle and the graphene substrate is simulated by a viscous damping force, which is proportional to the transport velocity. As the sliding block moves under the graphene substrate, a driving force is yielded due to the van der Waals interactions between the sliding block and the nanoparticle. The particle on the graphene substrate only moves when the driving force is larger than the in-plane force; as the particle moves, the damping force is spontaneously produced and replaces the in-plane force as a resistance barrier of the particle. Many simulations show that when the sliding block moves underneath the substrate and passes the nanoparticle, sometimes the nanoparticle on the graphene substrate can be driven to move forward with the sliding block, while sometimes it remains static. Typical movies are provided as Supplementary Movies 1 and 2. Figure 2 shows typical snapshots corresponding to the Supplementary Movies 1 and 2, where Fig. 2a,c show the initial states for the two cases and Fig. 2b,d denote the final states, respectively. In order to disclose the transport mechanism, the influencing factors of the driving force, in-plane force and the damping force will be investigated in detail.

### Effect of the particle’s size on the driving force and in-plane force

**Figure 3:**
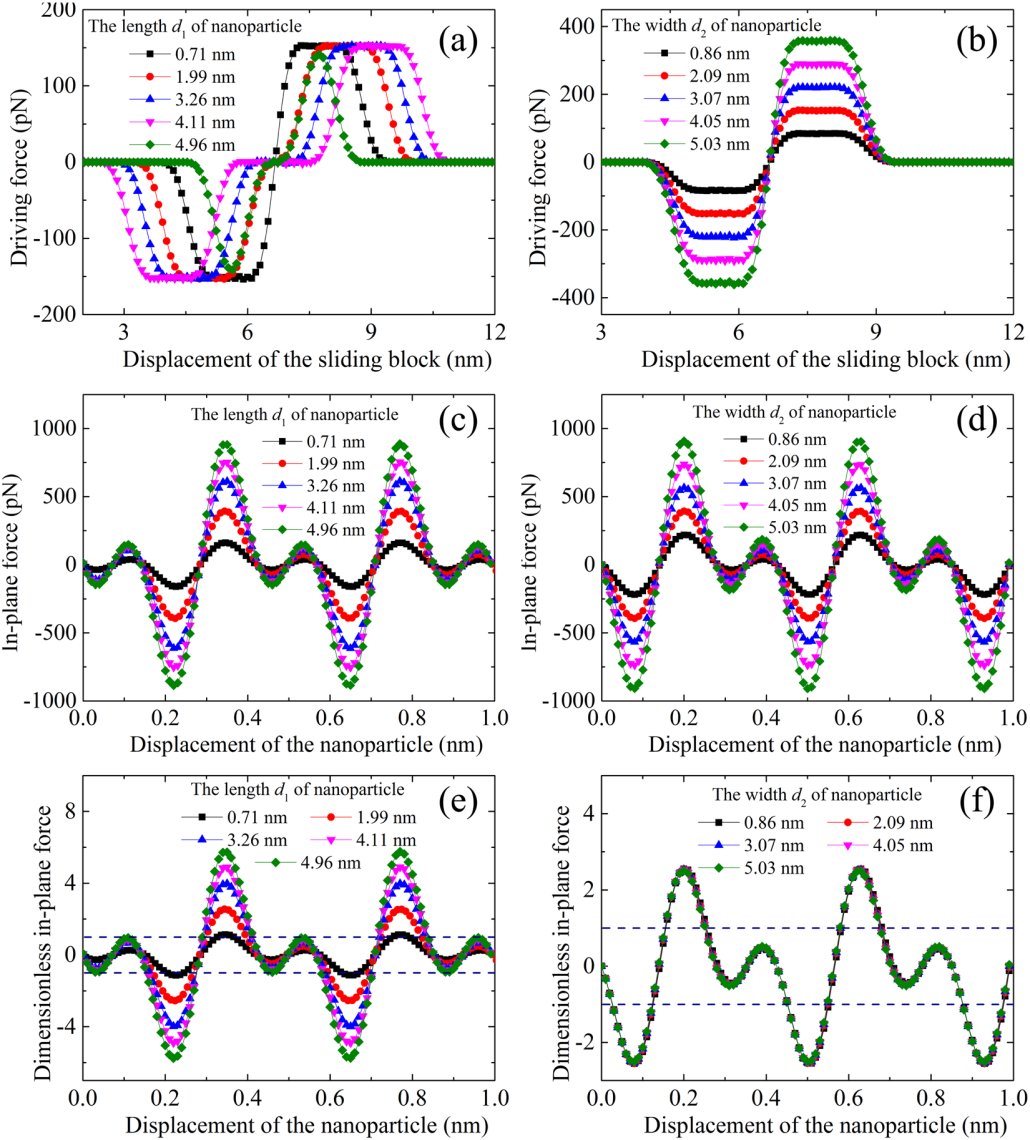
The effect of particle size on the driving force and the in-plane force. Driving force between the nanoparticle and the sliding block for (**a**) nanoparticles of different length but with a fixed 2.09-nm width, and for (**b**) nanoparticles of different width but with a fixed 1.99-nm length. In-plane force between the nanoparticle and the substrate for (**c**) nanoparticles of different length but with a fixed 2.09-nm width, and for (**d**) nanoparticles of different width but with a fixed 1.99-nm length. Dimensionless in-plane force for (**e**) nanoparticles of different length but with a fixed 2.09-nm width, and for (**f**) nanoparticles of different width but with a fixed a 1.99-nm length. The nanoparticle consists of a rectangular three-layered graphene sheet.

First, we consider the case of a nanoparticle consisting of a rectangular three-layered graphene sheet, which is fixed on the graphene sheet substrate. Similar to the typical example shown in Fig. 2, the sliding block moves from right to left and passes the nanoparticle. The driving force between the nanoparticle and the sliding block is achieved as shown in Fig. 3a,b for nanoparticles of different length but with a fixed 2.09-nm width and nanoparticles of different width but with a determined 1.99-nm length, respectively. The sign of the driving force indicates its direction, where a positive sign indicates the direction to the left, while a negative sign indicates the direction to the right. Both results in Fig. 3a,b demonstrate that the absolute value of the driving force gradually increases when the sliding block moves from the right to the left and approaches the nanoparticle. After achieving a maximum absolute value with the direction to the right, the absolute value of the force is gradually reduced to zero as the sliding block lies just below the nanoparticle. As the sliding block moves further to the left and away from the nanoparticle, the driving force increases gradually with a direction to the left and decreases gradually to zero after attaining a maximum. It means that the nanoparticle would have a right moving trend if the sliding block is located at its right side and would move to the left when the sliding block is located at its left side.

When the width *d*_2_ of the cross section is maintained at 2.09 nm and the length *d*_1_ is changed from 0.71 nm to 4.96 nm, the maximum driving force is hardly affected by *d*_1_, as shown in Fig. 3a. When *d*_1_ is maintained at 1.99 nm and *d*_2_ is changed from 0.86 nm to 5.03 nm, the maximum force increases, as shown in Fig. 3b. It means the maximum driving force is only influenced by the particle width.

The in-plane force between the nanoparticle and the graphene substrate will resist the initiation of the nanoparticle’s movement, which can be obtained by moving the nanoparticle on the graphene substrate. Figure 3c,d show the in-plane force of the nanoparticles consisting of a rectangular three-layered graphene sheet, where the effect of length and width of nanoparticles on the in-plane force is investigated. The in-plane force varies periodically from positive to negative values with a nearly zero average force, which is consistent with the finding in references^26,27^. It shows that the absolute value of the in-plane force generally increases with the increase in length and width of nanoparticles.

The initial movement of nanoparticles depends on the difference between the in-plane force and driving force. Figure 3e,f give the dimensionless in-plane force, which is nondimensionalized by the corresponding maximum driving force given in Fig. 3a,b, respectively. The two horizontal dotted lines in Fig. 3e,f correspond to 1 and −1. The absolute value of the dimensionless in-plane force generally increases with the increasing length of the nanoparticle. However, the dimensionless in-plane force is hardly affected by the width *d*_2_ of the nanoparticle, which is due to the proportional increase of in-plane force and driving force with the nanoparticle width *d*_2_ as shown in Fig. 3b,d.

In summary, the nanoparticle length *d*_1_ hardly influences the maximum driving force but significantly influences the in-plane force. The in-plane force of a nanoparticle with a relatively large length would be greater than its driving force, which, as a result, would prevent the movement of the nanoparticle. However, the nondimensionalized in-plane force by the corresponding maximum driving force is hardly influenced by the nanoparticle width *d*_2_, although both of them increase with the increasing particle width *d*_2_. Therefore, the driving behavior of a nanoparticle on a graphene substrate without pre-tension strain is mainly affected by its length *d*_1_.

In the following text, we mainly focused on an approximately square nanoparticle, whose side length *d* is defined as the average value of its length *d*_1_ and width *d*_2_.

**Figure 4:**
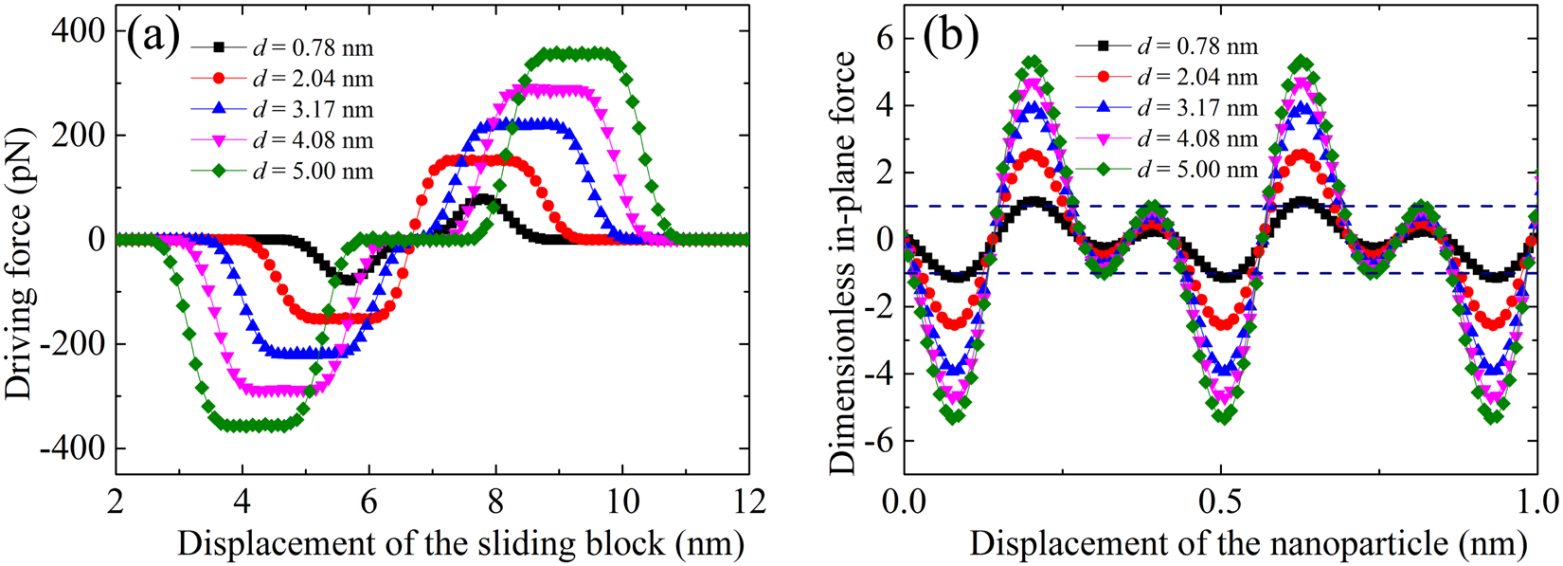
The effect of the average side length of square nanoparticles on (**a**) driving force and (**b**) dimensionless in-plane force.

Figure 4a,b show the effect of the average side length of a square nanoparticle on the driving force and on the nondimensionalized in-plane force by the corresponding maximum driving force, respectively. The horizontal lines are also shown in Fig. 4b. Both the driving force and the dimensionless in-plane force increase with the increasing side length of nanoparticle. When the side length of the nanoparticle is not less than 2.04 nm, the maximum in-plane force will be much larger than the maximum driving force, while the driving force of the nanoparticle with a side length of 0.78 nm is nearly equal to the in-plane force. It means that square nanoparticles with a side length less than 0.78 nm would be driven to move with the sliding block, while square nanoparticles with a side length larger than 2.04 nm would not.

The influence of nanoparticle thickness and sliding block length on both forces have also been considered, both of which have no obvious effect on the maximum driving force and in-plane force. Detailed discussions can be found in Figs S1 and S2 of Supplementary Material.

### Effect of pre-tension of the substrate on the in-plane force

**Figure 5:**
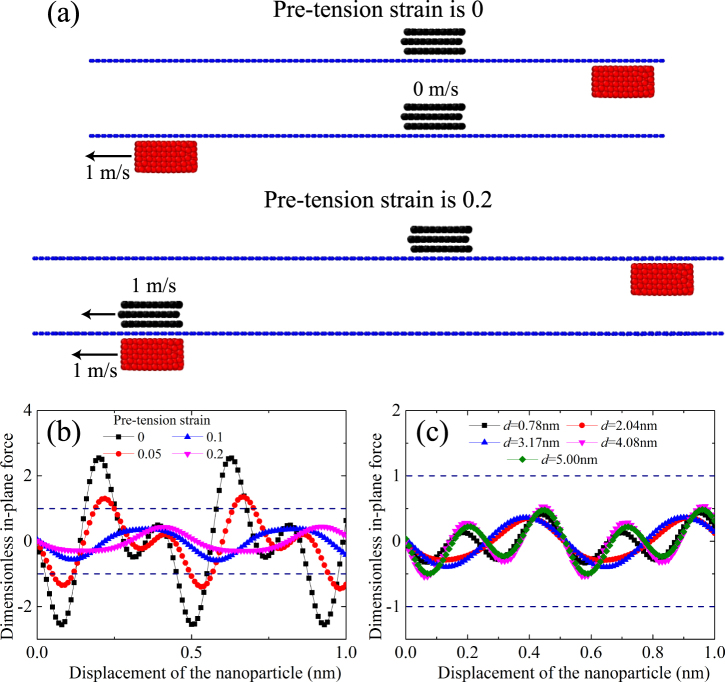
The effect of pre-tension strain in the substrate on the driving behavior. (**a**) Schematic of the driving behavior with and without a pre-tension strain. The dimensionless in-plane force between the nanoparticles and graphene substrates (**b**) for graphene substrates with different pre-tension strains, whereby the nanoparticle is an approximately square three-layered graphene sheet with an average side length of 2.04 nm, and (**c**) for square three-layered graphene sheet nanoparticles with different average side lengths and a 0.2 pre-tension strain in the graphene substrate.

As discussed above, the in-plane force of a large nanoparticle is always larger than the driving force, preventing the initial movement of nanoparticle. The question of how to drive a relatively large nanoparticle is put forward. Inspired by a study demonstrating that the tension strain in a graphene sheet could change the wettability of a surface based on a change in the atomistic lattice^28^, we adopt the novel idea to study the possible motion of large particles. Cases of graphene substrate with and without pre-tension are analyzed. If a relatively large nanoparticle is put on a graphene substrate without pre-tension, both initial and final states of the nanoparticle on the graphene substrate is similar to Fig. 2c,d. When the graphene substrate is pre-tensioned with 0.2 strain, the results are illustrated in Fig. 5 for the nanoparticle consisting of square three-layered graphene with an average side length of 2.04 nm. It was found that the pre-tension strain in the graphene substrate significantly influences the transport behavior of the nanoparticle. Without any pre-tension strain in the graphene substrate, the nanoparticle does not move as the sliding block moves from right to left at 1 m/s. However, the nanoparticle moves with the sliding block at the same speed of 1 m/s when a pre-tension strain of 0.2 is added at the graphene substrate as shown in Fig. 5a.

The pre-tension strain in the graphene substrate does not show any influence on the driving force between the nanoparticle and the sliding block, details of which can be found in Fig. S2b in Supplementary Material. The only possible change should occur for the in-plane force. The in-plane force between the nanoparticle and the graphene substrate is calculated for different transport systems, as shown in Fig. 5b,c, where the in-plane force is non-dimensionalized by the respective maximum driving force.

Figure 5b gives the dimensionless in-plane force, where the absolute value of the in-plane force in the pre-tension systems is much smaller than that in the system without pre-tension. Furthermore, both the absolute value of the in-plane force and the maximum in-plane force decrease with the increasing pre-tension strain in the graphene substrate. This result is consistent well with previous findings^26,29^, whereby a commensurate or incommensurate state was achieved by placing a graphene flake on a graphene substrate with an *AB* stacking orientation or with a graphene flake rotation of 90°. In the present model, an analogous incommensurate state is attained by stretching the graphene substrate. When the pre-tension strain in the graphene substrate vanishes, the amplitude of the in-plane force reaches the maximum and becomes larger than the maximum driving force. As a result, the nanoparticle cannot be driven and remains still. When the pre-tension strain in the graphene substrate is larger than 0.1, the amplitude of the in-plane force becomes smaller than the maximum driving force. Consequently, the nanoparticle is driven to move. A series of simulations demonstrate that a pre-tension strain above ~0.1 is needed in the graphene substrate to ensure transport of nanoparticles.

Figure 5c shows the dimensionless in-plane force between the graphene substrate with a pre-tension strain of 0.2 and the nanoparticle consisting of a square three-layered graphene sheet with different side lengths. It is found that the absolute value of the in-plane force in the pre-tension systems is much smaller than the corresponding driving force, and the dimensionless maximum in-plane force is approximately irrelevant to the side length of nanoparticles. As a result, nanoparticles with side lengths even larger than 5.00 nm can be driven on the pre-tensioned graphene substrate by the sliding block.

### Effect of sliding velocity on the transport behavior

**Figure 6:**
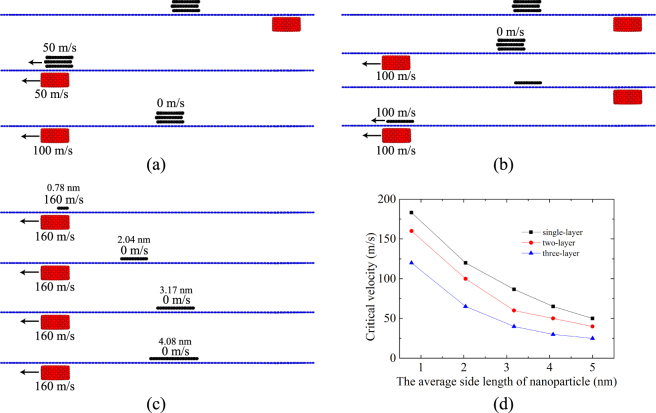
The effect of (**a**) the sliding velocity of the sliding block, (**b**) the number of layers in the nanoparticle and (**c**) the average side length of the nanoparticle. (**d**) The critical velocity as a function of the average side length and the number of layers for the nanoparticle with *γ* = 0.03 eVps/nm^2^.

Based on the comparison of the driving force and in-plane force, we find that only small nanoparticles could be moved on the graphene substrate without pre-tension, while almost all nanoparticles could be moved on the pre-tensioned graphene substrate with a pre-tension strain of 0.2. Further simulations show that transport is also influenced by the sliding velocity of the sliding block, the number of layer and the average side length of nanoparticle, which is briefly illustrated in Fig. 6.

Figure 6a shows the influence of the sliding velocity of the sliding block on the driving behavior of the nanoparticle. Initially, the sliding block is at the right end of the substrate with a pre-tension strain of 0.2. When the sliding block moves from the right to the left at a speed of 50 m/s, the nanoparticle moves forward with a similar velocity. However, when the sliding block moves from the right to the left at a speed of 100 m/s, the nanoparticle moves initially and is then left behind the sliding block. Finally, the nanoparticle stops at the position shown in the bottom picture of Fig. 6a, which is due to the viscous damping force that increases with the velocity of the nanoparticle. At some point, the damping force becomes larger than the driving force, decreasing the velocity of the nanoparticle. Consequently, the driving force vanishes when the sliding block moves away from the nanoparticle. A series of simulations demonstrate that the critical sliding velocity of the sliding block to enable successful transport is about 65 m/s. Below the critical sliding velocity, the nanoparticle would be driven forward definitely by the sliding block and finally achieve a steady moving state with the sliding block.

Further simulations show that the driving behavior is also influenced by the side length and thickness of the nanoparticles. The driving behavior of single-layered and three-layered nanoparticles is typically considered, the initial state and the final state of which are illustrated in Fig. 6b, respectively. Here, by tuning the number of layers, we tune the thickness of the nanoparticles, i.e., a three layered nanoparticle is thicker than a single-layered nanoparticle. The single-layered nanoparticle could be driven to move forward but the three-layered one is left behind when the sliding block moves from right to left at a velocity of 100 m/s. As mentioned earlier, the driving force and in-plane force are almost irrelevant to the number of layers of the nanoparticle, but the dynamic friction simulated by the viscous damping force would increase with the increase of the atoms of nanoparticles and the moving velocity. As a result, a large nanoparticle will be driven to move a small distance and then stop because of the increasing damping force.

Similar to the effect of the thickness of the nanoparticle, the driving behavior is also influenced by the side length of the nanoparticles, as shown in Fig. 6c. As the sliding block moves from the right to the left at a constant velocity of 160 m/s, only the smallest nanoparticle (average side length of 0.78 nm) is captured and moves along with the sliding block. The nanoparticle with an average side length of 2.04 nm moves forward a short distance and then stops while the larger particles with average side lengths almost stay still. All these phenomena are due to the competition between the driving force and the viscous damping force. For a relatively large particle on a pre-tensioned graphene substrate, the nanoparticle starts to move because driving force is larger than the in-plane force, as shown in Fig. 6c; however, the viscous damping force may increase sharply with the moving velocity of the nanoparticle, which in turn reduces the velocity of the nanoparticle itself. When the sliding block moves away from the nanoparticle at a high speed, the driving force quickly vanishes. As a result, the large nanoparticle stops after a distance or remains at the initial position.

The critical sliding velocity of the sliding block, below which nanoparticles of different side lengths and with different numbers of layers could be driven by the sliding block, is given in Fig. 6d after a series of simulations. Sliding block with a relatively low sliding velocity could be helpful for the movement of nanoparticles. The shorter the side length of the nanoparticle or the smaller the thickness of the nanoparticle, the larger the critical velocity of the sliding block is permitted.

### Effect of viscous damping force on the transport behavior

The transport behavior of nanoparticles without viscous damping force is also considered, in which the kinetic energy of the nanoparticle induced by the sliding block is difficult to be dissipated. The captured nanoparticle will move back and forth around the sliding block as shown in Fig. S3a of Supplementary Material. The nanoparticle left behind would vibrate with a relatively long time to dissipate the energy, as shown in Fig. S3b. Typical movies are also shown as Supplementary Movies 3 and 4.

For possible applications in the future, we should consider the pre-existing wrinkles or defects in the substrate surfaces, which would increase the friction force^30^. Furthermore, at a high velocity, experimental observations also show that the friction value may be higher^31^. In our simulation, the substrate is assumed to be atomically smooth, which may obviously reduce the dissipation of kinetic energy. The “hade-make” viscous damping force is adopted in order to approach cases in real applications. With the damping force, the kinetic energy of the nanoparticle can be quickly dissipated. As a result, the moving nanoparticle will be tightly captured by the sliding block or the nanoparticle left behind could stop at the equilibrium position without large vibration back and forth.

**Figure 7:**
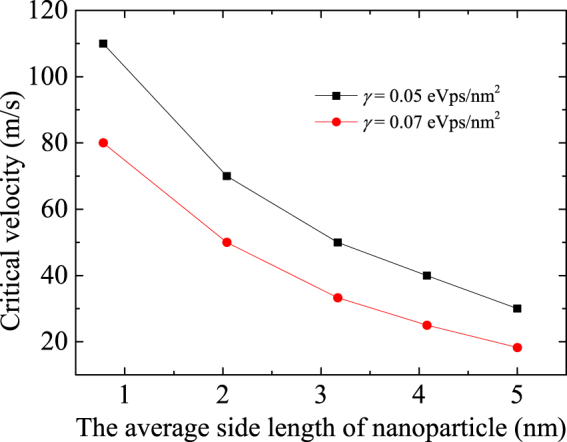
Critical velocity as a function of the average side length of an approximately square nanoparticle for *γ* = 0.05 and 0.07 eVps/nm^2^.

In all the above simulations, the viscous damping coefficient is *γ* = 0.03 eVps/nm^2^, which should influence the damping force of a moving nanoparticle. Different damping coefficients are adopted in the simulations (e.g., 0.05 and 0.07 eVps/nm^2^). The transport behavior is similar to that when the damping coefficient is 0.03 eVps/nm^2^; the only difference is the permitted critical velocity. Figure 7 gives the corresponding critical velocity as a function of the length of the nanoparticles for *γ* = 0.05 and 0.07 eVps/nm^2^, where the critical velocity decreases with an increasing damping coefficient.

### Effect of temperature and interface commensurability on the transport behavior

All the above simulations are carried out at 1 K, which can also be done at a room temperature (300 K). In addition, the effect of the initial interface commensurability on the transport behavior is also investigated. Details of both kinds of effects can be found in Supplementary Material. It shows that the general transport mechanism of nanoparticles is not influenced by the system temperature, however, nanoparticles would perform a small thermal vibration around the equilibrium position in the system at room temperature, similar to the Brownian motion in Jafary-Zadeh *et al*.^32^. A small nanoparticle on a commensurate interface may have a much larger in-plane force than a large nanoparticle on an incommensurate interface if the substrate is not pre-tensioned. However, when the substrate is stretched with pre-tension strain, the interface will be always incommensurate. The effect of interface commensurability on the transport behavior of nanoparticles is much weaker than the effect of pre-tension.

Copper material is used for the sliding block in all simulations. Similar driving behaviors would also occur using gold, nickel, or other metal sliding blocks.

### Application of the technique to design a nano-sieve

**Figure 8:**
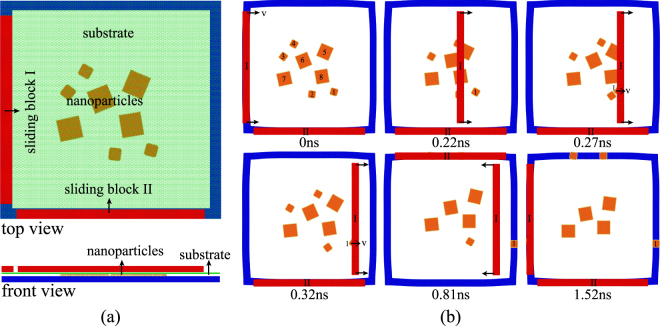
The schematic of a nano-sieve. (**a**) Initial configuration of a nano-sieve model, where the two perpendicular red bars are sliding blocks and eight square single-layered graphene nanoparticles with average side lengths of 2.04 and 4.08 nm are randomly distributed on the graphene substrate. (**b**) Snapshots of the separation process, the two perpendicular blocks slide at a speed of 100 m/s.

The above technique is further adopted to study the separation of nanoparticles of different sizes but with the same shape. The initial configuration and typical snapshots of the nano-sieve are illustrated in Fig. 8. Eight square nanoparticles are randomly distributed on a graphene substrate with pre-tension strain. The average side lengths of nanoparticles are 2.04 and 4.08 nm, while the side length of the graphene substrate is 40 nm. The boundary of each sheet is saturated by hydrogen atoms to avoid aggregation^33^. Two perpendicular sliding blocks under the graphene substrate are used to enable more efficient separation.

When the two sliding blocks move reciprocally at 100 m/s several times, the smaller nanoparticles with a side length of 2.04 nm are more easily removed, as shown in Fig. 8b, and the larger nanoparticles with a side length of 4.08 nm remain on the substrate. All the smaller nanoparticles are removed to the boundaries of the substrate after several reciprocal movements of the sliding blocks, typical movies are also provided as Supplementary Movie 5.

The effect of the sliding velocity of the block on the separation of nanoparticles of different sizes is shown in Fig. S6. When a sliding velocity of 60 m/s is adopted, all the nanoparticles are cleaned from the substrate after several reciprocal movements of the sliding blocks, as shown in Fig. S6a. However, all the nanoparticles will approximately keep still no matter how many times of the reciprocal sliding if the velocity of the sliding blocks is 150 m/s, as shown in Fig. S6c. An appropriate velocity of 100 m/s would ensure successful selection of the two kinds of nanoparticles, as shown in Fig. S6b.

## Conclusions

Nanoparticles have increasing applications. Transporting and separating nanoparticles have attracted many interesting investigations. In this paper, a new transport technique is proposed based on the van der Waals interaction between nanoparticles and a sliding block. The results show that if the in-plane force between nanoparticles and the substrate is less than or equal to the driving force between the nanoparticles and the sliding block, nanoparticles can begin to move. The subsequent movement of nanoparticles depends mainly on the competition between the driving force and the damping force. The pre-tension in the graphene substrate could reduce the in-plane force, which would be helpful for nanoparticle transport. Effects of nanoparticle sizes and thickness, as well as the speed of the sliding blocks on the transport behavior are investigated in detail. The nanoparticle transport would be easier if the speed of the sliding block is lower. A critical velocity is put forward as a key parameter to describe the transport behavior, below which the nanoparticle can be driven. Generally, the critical velocity decreases with the increase of nanoparticle size and thickness, as a result, transport is easier for smaller and thinner nanoparticles. Furthermore, the transport would also be easier with a lower viscous damping force and a higher temperature. The initial interface commensurability has no obvious effect on the transport behavior, especially in the case with a pre-tensioned substrate. The present technique can be further applied to design a novel nano-sieve, with which nanoparticles of different sizes can be selected.

## Methods

### The driving model

In Fig. 1, the AIREBO potential^34^ is used to describe two different types of interactions: (1) interactions between carbon (C) atoms in the graphene nanoparticle, and (2) interactions between carbon atoms in the graphene substrate. The interactions between copper (Cu) atoms in the sliding block abide by the EAM potential^35^. The interactions between C atoms in the graphene nanoparticle and C atoms in the graphene substrate abide by the Lennard-Jones potential $V(r)=4\varepsilon ({\sigma }^{12}/{r}^{12}-{\sigma }^{6}/{r}^{6})$ with *ε* = 2.968 meV and *σ* = 0.3407 nm^36^. Similarly, the interaction between C atoms in the graphene nanoparticle and Cu atoms in the sliding block also abides by the Lennard-Jones potential but with *ε* = 19.996 meV and *σ* = 0.3225 nm^37^. The cut-off distance and the time step in all the simulations are set as 1 nm and 1 fs, respectively. We used the open-source Large-scale Atomic/Molecular Massively Parallel Simulator (LAMMPS) software for all simulations, and the temperature was fixed at 1 K with a NVT ensemble^38^.

### The nano-sieve model

In Fig. 8, the potentials to describe the interactions between C-C, C-Cu, and Cu-Cu atoms are the same as those used for the system shown in Fig. 1. The REBO potential is adopted to describe the C-H interaction in the nanoparticles; the interactions between hydrogen atoms and other atoms in the substrate and sliding blocks are negligible.

## Electronic supplementary material

Supplementary Material

Supplementary Movie 1

Supplementary Movie 2

Supplementary Movie 3

Supplementary Movie 4

Supplementary Movie 5

Supplementary Movie 6

Supplementary Movie 7
