# Supplementary material for: A new technique for nanoparticle transport and its application in a novel nano-sieve
Source: Sci Rep. 2018 Jun 26;8:9682. doi: 10.1038/s41598-018-28033-5 (PMC6018662; doi:10.1038/s41598-018-28033-5)
Supplement: Supplementary file 1 — Supplementary Material [file 41598_2018_28033_MOESM1_ESM.pdf]

Supplementary Material

A new technique for nanoparticle transport and its application in a novel nano-sieve

Shuai Wang [a, d] Chao Wang [a] Zhilong Peng [b, c] Shaohua Chen [b, c, 1]

[a] LNM, Institute of Mechanics, Chinese Academy of Sciences, Beijing 100190, China

[b] Institute of Advanced Structure Technology, Beijing Institute of Technology, Beijing, 100081, China

[c] Beijing Key Laboratory of Lightweight Multi-functional Composite Materials and Structures, Beijing Institute of Technology, Beijing, 100081, China

[d] School of Engineering Sciences, University of Chinese Academy of Sciences, Beijing 100049, China

S1. Methodology

The system is relaxed first with a fixed substrate to obtain the realistic equilibrium interatomic distance and configuration. After the system relaxation, atoms at both ends of the substrate move to the opposite direction to induce the substrate pre-tension strain. Finally, the system is relaxed again to achieve a pre-tensioned graphene substrate. The sliding block can be controlled to move from right to left beneath the graphene substrate with or without pre-tension strain. In all the simulations, the graphene substrate is assumed to be smooth.

S2. The driving force and the in-plane force

[1] Corresponding author. E-mail: chenshaohua72@hotmail.com or shchen@bit.edu.cn

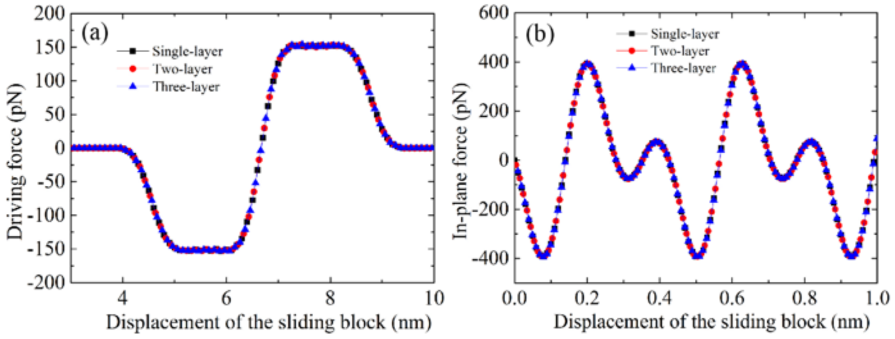

Figure S1. The effect of nanoparticle thickness on (a) the driving force and (b) the in-plane force. The average side length of nanoparticle is 2.04 nm.

Besides the effect of particle’s size on the driving force and the in-plane force in the main text, the influence of nanoparticle thickness and sliding block length is also discussed. Simulations show that the thickness of the nanoparticle hardly influences the driving force between the nanoparticle and the sliding block as well as the in-plane force between the nanoparticle and the substrate, as shown in Fig. S1, which is mainly due to the small effective interacting distance and the cut-off distance.

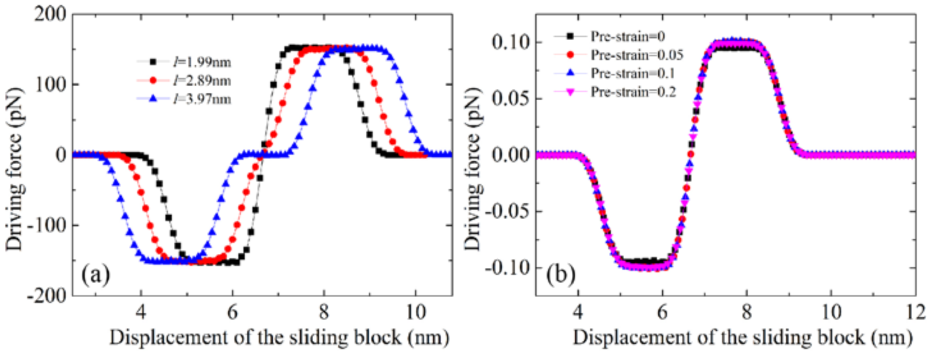

Figure S2. Driving force between a three-layered graphene nanoparticle and a sliding block for (a) different sized sliding block and (b) substrate with different pre-strain. The nanoparticle consists of an approximately square three-layered graphene sheet with a 2.04-nm side length.

The effect of the length of the sliding block on the driving force is shown in Fig. S2a, where the average side length of the nanoparticle consisting of an approximately square three-layered graphene sheet is fixed as 2.04 nm. It shows that the maximum driving force is not influenced by the length of the sliding block, and only the location that the maximum driving force emerges is different. If the length of the sliding block is short enough, it would influence the maximum driving force, which decreases as the length of the sliding block decreases. However, a phase transition would occur if the cross-sectional area is too small[1]; Such a case is not considered in the present work. A series of simulations shows that the maximum driving force is almost insensitive to the length of the sliding block when it is larger than 2.04 nm.

Further simulations show that the driving force between the nanoparticle and the sliding block is hardly influenced by the pre-tension strain of substrate, as shown in Fig. S2b. It should be noted that the pre-tension strain is a major influencing factor of the in-plane force between the nanoparticle and substrate, which is discussed in detail in the main text.

S3. The effect of viscous damping force and temperature

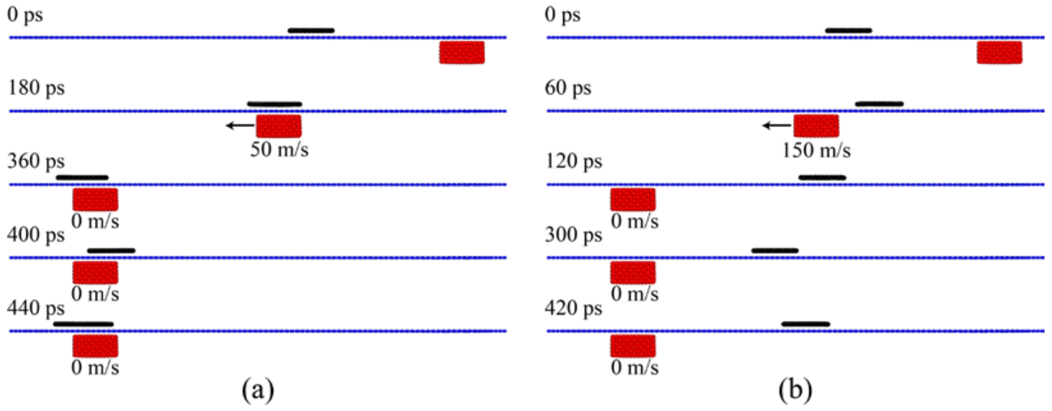

Figure S3. The transport behavior of the nanoparticle without the viscous damping force. The sliding block moves 18 nm under the substrate with a sliding velocity of (a) 50 m/s and (b) 150 m/s, and then the system is relaxed for a while with the sliding block fixed. The average side length of nanoparticle is 2.04 nm, the temperature is fixed at 1 k.

A viscous damping force is used to improve the dynamic friction force in the main text, by which the kinetic energy of the nanoparticle can be easily dissipated. The transport behavior of nanoparticle without the viscous damping force is also considered, as illustrated in Fig. S3. When the sliding block moves under the substrate at a speed of 50 m/s, the nanoparticle could be captured by the sliding block, however, the kinetic energy of the nanoparticle is difficult to dissipate. As a result, the nanoparticle will move back and forth around the sliding block, as shown in Fig. S3a. The nanoparticle will be left behind and vibrate with a relatively long time to dissipate the energy when the sliding block moves at a speed of 150 m/s, as shown in Fig. S3a. Typical movies are also provided as Supplementary Movies 3 and 4.

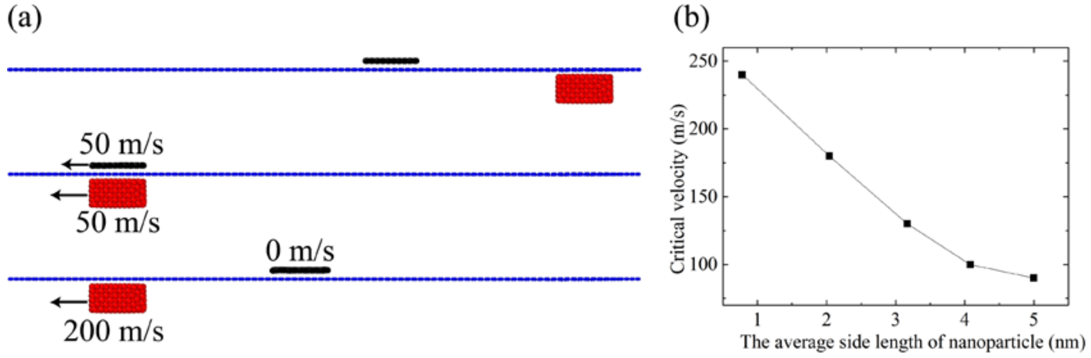

Figure S4. The transport behavior of the nanoparticle at a room temperature (300K). (a) Schematic of the driving behavior with different sliding velocities of the sliding block, the average side length of nanoparticle is 2.04 nm. (b) The critical velocity as a function of the side length at a room temperature (300K).

All the simulations can be carried out at a room temperature. The transport behavior of the nanoparticle with different sliding velocities of the sliding block is illustrated in Fig. S4a. Similar to the transport behavior at 1k, the nanoparticle can be driven to move forward by the sliding block with a lower sliding velocity, but left behind in the case with the sliding block at a higher sliding velocity. Furthermore, the nanoparticle may perform a small thermal vibration around the equilibrium position as shown in Supplementary Movies 6 and 7, which is similar to the Brownian motion in Jafary-Zadeh et al.[2]. As shown in Fig. S4b, the critical velocity of the sliding block at the room temperature decreases also with an increasing side length of the nanoparticle, but is larger than the corresponding value at 1k as shown in Fig. 6d.

S4. The effect of the interface commensurability on the transport behavior

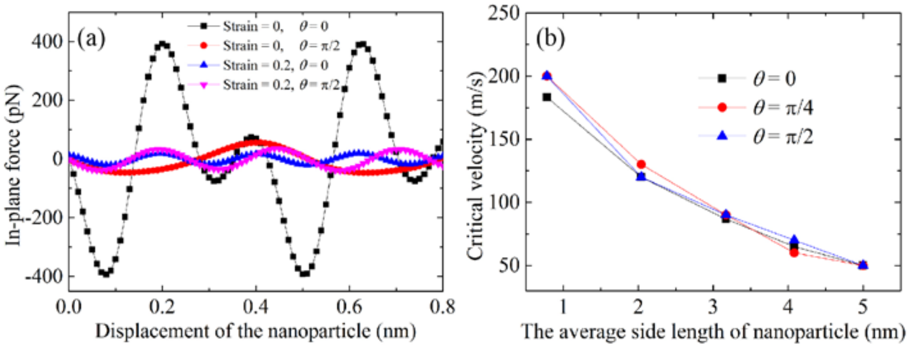

Figure. S5. The effect of initial commensurability on the driving behavior. (a) The in-plane force between the nanoparticle and substrate with different initial interface commensurability and pre-tension strain, the average side length of nanoparticle is 2.04 nm. (b) The critical velocity of the sliding block with different initial interface commensurability and average side length of the nanoparticle with 0.2 pre-tension strain in the substrate. θ is the rotation angle of nanoparticle from *AB* stacking orientation.

The effect of initial interface commensurability on the transport behavior has also been investigated. As shown in Fig. S5, θ is the rotation angle of nanoparticle from *AB* stacking orientation, the initial interface between nanoparticle and substrate is commensurate with $\theta = 0$, but incommensurate with $\theta = \pi/2$. The average particle side length of 2.04 nm is adopted as an example to illustrate the effect of interface commensurability and pre-tension strain on the in-plane force as shown in Fig. S5a. It shows that on a substrate without pre-tension strain, the in-plane force of nanoparticle on an incommensurate interface is more than ten times than that on a commensurate interface. Therefore, it is possible that a small nanoparticle on a commensurate interface may have a much larger in-plane force than a large nanoparticle with an incommensurate interface. However, when the substrate is stretched with pre-tension strain, the interface will be always incommensurate. The effect of interface commensurability on transportation and selection of particles is much weaker than the effect of pre-tension, which can be found in Fig. S5a, where the in-plane force is given for both commensurate and incommensurate cases with pre-tension strain in the substrate.

We further check the critical velocity in cases with different initial commensurability as shown in Fig. S5b, since the critical velocity is a key parameter to determine the transport behavior and size-selection of particles. It is found that the commensurate or incommensurate interface between the nanoparticle and substrate has no significant influence on the critical velocity.

S5. Application of the technique to design a nano-sieve

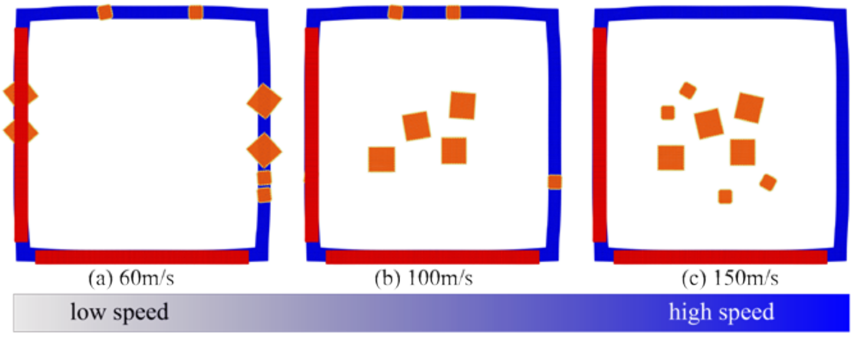

Figure S6. Transport results achieved at three different sliding velocities.

The effect of the sliding velocity of the block on the separation of nanoparticles of different sizes is shown in Fig. S6, in which all the nanoparticles can be cleaned, keep still or selected by different sliding velocity.

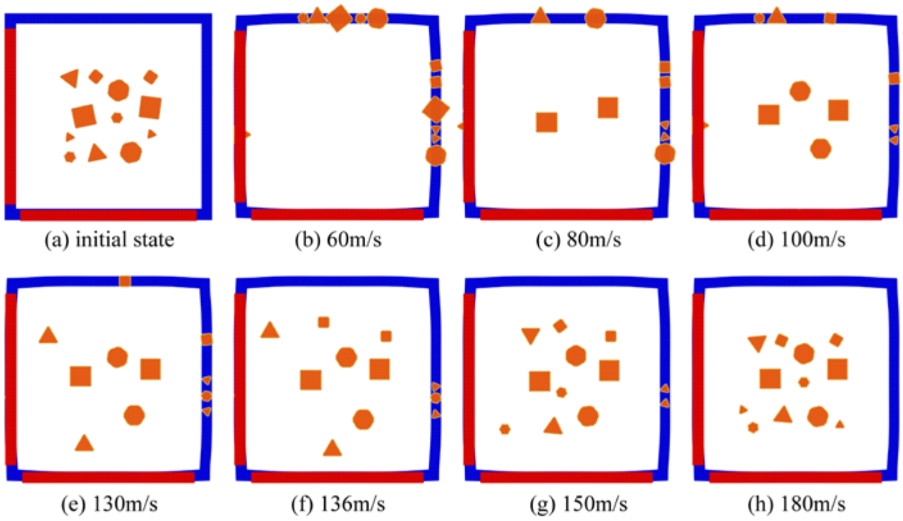

Figure S7. Transport behavior of nanoparticles of different sizes and shapes using the sliding blocks with different sliding speeds.

In reality, the nanoparticles may have different shapes, which may also influence the transport behavior. The separation of nanoparticles with different sizes and shapes is shown in Fig. S7, including square, circular and triangular nanoparticles. The size of the nanoparticle is about 2 nm and 4 nm, which is defined as the side length of a square nanoparticle, the

diameter of a circular nanoparticle or the side length of an equilateral nanoparticle. All the nanoparticles are distributed randomly on the graphene substrate. After several reciprocal movements of two perpendicular sliding blocks at 60 m/s or 180 m/s, all nanoparticles can be swept off the substrate or left behind, as shown in Figs. S7b and h. Furthermore, a part of nanoparticles could be swept off while different sliding velocities are adopted. For example, only two square nanoparticles with size about 4 nm are left if the sliding velocity is 80m/s as shown in Fig. S7c. While both square and circular nanoparticles with size about 4 nm left behind while a sliding velocity of 100 m/s is adopted. The critical velocity of nanoparticle with different shapes and sizes are illustrated in Fig. S8, which could be used to explain the selection behavior shown in Fig. S7. If the size of particle keeps constant, the critical velocity of a circular nanoparticle is lower than that of an equilateral triangular nanoparticle but larger than that of a square one. As a result, the circular particle is easier to be swept off than a square one but difficult than an equilateral triangular one, which is in accordance with the phenomenon shown in Figs. S7c-e for nanoparticle with size of 4 nm and the phenomenon shown in Figs. S7f-h for nanoparticle with size of 2 nm.

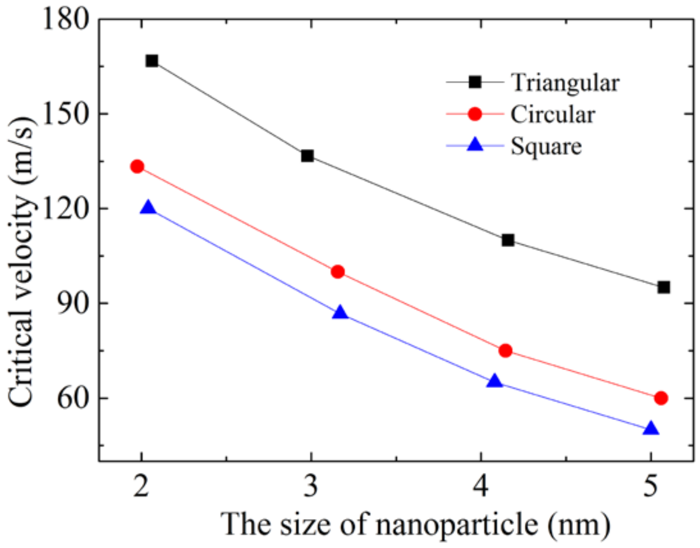

Figure S8. The critical velocity of the sliding block for different particle sizes and shapes.

Such a technique could also be applied to remove impurities embedded inside a nano-device. An example based on a carbon nanotube is given in Fig. S9, in which impurities inside a carbon nanotube can be cleaned using the present technique.

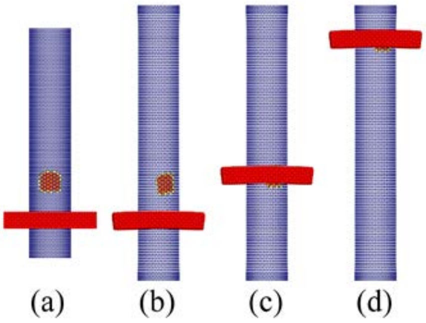

Figure S9. An impurity inside of a carbon nanotube is cleaned using a velocity of 100 m/s for the ring sliding block: (a) the initial state, (b) pre-tension state, and (c-d) snapshots of the driving process at 20 and 150 ps.

References

1. Diao, J., Gall, K. & Dunn, M. L. Surface-stress-induced phase transformation in metal nanowires. *Nat Mater* **2,** 656–660 (2003).

2. Jafaryzadeh, M., Reddy, C. D., Sorkin, V. & Zhang, Y. W. Kinetic nanofriction: a mechanism transition from quasi-continuous to ballistic-like Brownian regime. *Nanoscale Res. Lett.* **7,** 1–8 (2012).
